# Polyglycerol-Shelled Reduction-Sensitive Polymersome for DM1 Delivery to HER-2-Positive Breast Cancer

**DOI:** 10.1021/acs.biomac.4c00512

**Published:** 2024-06-22

**Authors:** Guoxin Ma, Daniel Braatz, Peng Tang, Yian Yang, Elisa Quaas, Kai Ludwig, Nan Ma, Huanli Sun, Zhiyuan Zhong, Rainer Haag

**Affiliations:** † Institut für Chemie und Biochemie, Freie Universität Berlin, Takustr. 3, Berlin 14195, Germany; ‡ Institute of Active Polymers, Helmholtz-Zentrum HEREON, Teltow 14513, Germany; § Biomedical Polymers Laboratory, College of Chemistry, Chemical Engineering and Materials Science, and State Key Laboratory of Radiation Medicine and Protection, Soochow University, Suzhou 215123, PR China

## Abstract

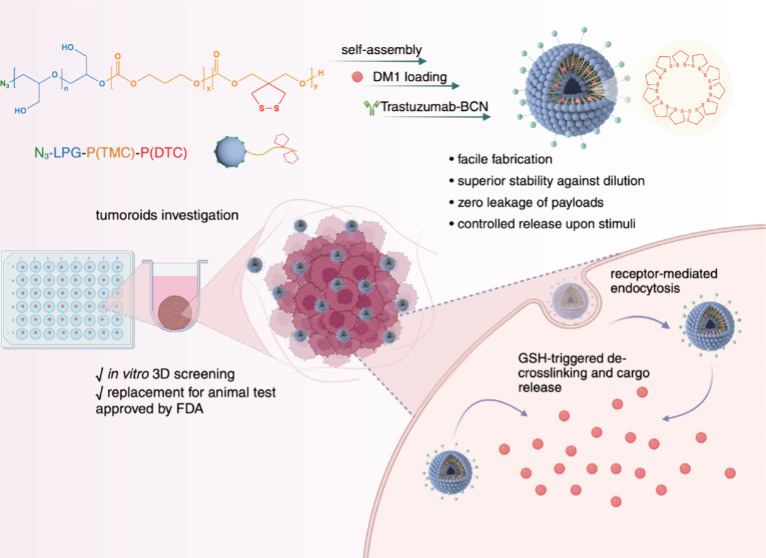

Supramolecular delivery systems with the prolonged circulation, the potential for diverse functionalization, and few toxin-related limitations have been extensively studied. For the present study, we constructed a linear polyglycerol-shelled polymersome attached with the anti-HER-2-antibody trastuzumab. We then covalently loaded the anticancer drug DM1 in the polymersome via dynamic disulfide bonding. The resulted trastuzumab-polymersome-DM1 (Tra-PS-DM1) exhibits a mean size of 95.3 nm and remarkable drug loading efficiency % of 99.3%. In addition to its superior stability, we observed the rapid release of DM1 in a controlled manner under reductive conditions. Compared to the native polymersomes, Tra-PS-DM1 has shown greatly improved cellular uptake and significantly reduced IC_50_ up to 17-fold among HER-2-positive cancer cells. Moreover, Tra-PS-DM1 demonstrated superb growth inhibition of HER-2-positive tumoroids; specifically, BT474 tumoroids shrunk up to 62% after 12 h treatment. With exceptional stability and targetability, the PG-shelled Tra-PS-DM1 appears as an attractive approach for HER-2-positive tumor treatment.

## 1 Introduction

Breast cancer is the most frequently diagnosed malignancy as well as the leading cause of death among women.^1,2^ It is well established that the overexpression of HER-2 (human epidermal growth factor receptor 2) is associated with prognosis and aggravation of breast cancer.^3−5^ Notably, HER-2 is amplified between 20- and 50-fold in multiple cancer types, causing aggressive tumor progression.^6−8^ The overexpression of HER-2 stands alongside tumor size, type, and stage as a critical index for tumor evaluation.^9^ Therefore, therapeutic monoclonal antibodies targeted against HER-2 are under widespread clinical development and investigation.^10−12^ ADCs (antibody-drug conjugates)^13,14^ and PDCs (polymer-drug conjugates)^15−17^ have been developed to improve targetability and reduce the systemic toxicity induced by traditional chemotherapeutics. Yet, these conjugates’ poor stability in circulation and their limited drug-loading capacity pose substantial obstacles to their clinical efficacy. To realize greater mechanical stability and more efficient shielding for active toxins, recent studies have exploited a large variety of supramolecular carriers as vehicles, including micelles,^18,19^ polymeric vesicles,^20,21^ and others. Polymersomes have attracted abundant interests in drug transportation by virtue of their extraordinary stability against dilution and their flexibility in carrying a wide array of payloads.^22^

Generally, polymeric micelles and polymersomes are fabricated from amphiphilic copolymers. Polyethylene glycol (PEG), considered a safe and biocompatible hydrophilic polymer, is widely used as a hydrophilic component for self-assembled carriers.^23,24^

In the past, we have developed diverse supramolecular systems assembled from PEG-poly trimethylene-carbonate (PTMC) and its dithiolaned derivative (PDTC) block copolymer for drug delivery. A well-defined structure, superior stability in circulation, stimuli-responsive degradation, and extraordinary efficacy in vivo of these systems are reported in our previous studies.^25−27^ However, many recent studies have addressed the risks of PEG, such as inciting PEG antibodies, accelerating blood clearance, and inciting sustained immunological reactions.^28−30^ Polyglycerol (PG), exhibiting stronger hydrophilicity^31−34^ and longer half-time than PEG,^35^ has emerged as an expedient alternative for PEG in biomedical and pharmaceutical areas.

**Scheme 1:**
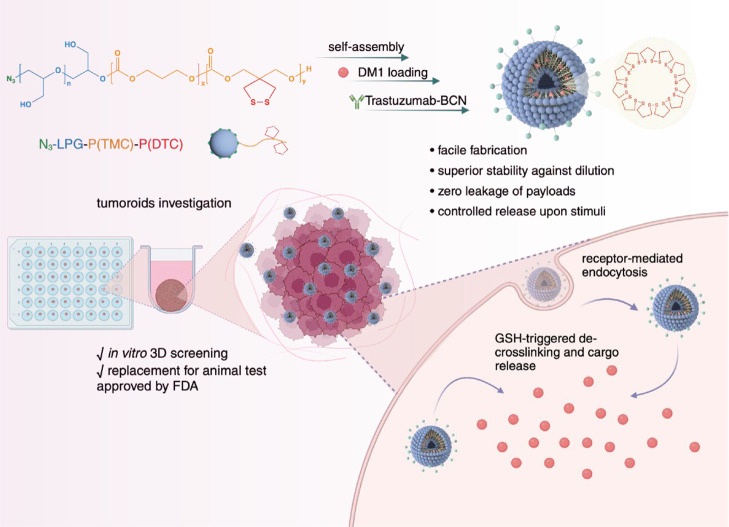
Illustration of Trastuzumab-Functionalized PG-Polymersome Loaded with DM1 (Tra-PS-DM1) for HER-2-Positive Breast Cancer Therapy, from Synthesis to 3D Tumoroid Screening

Aiming to develop a HER-2-targeting polymersome to deliver DM1 for breast cancer therapy, we therefore designed an amphiphilic block copolymer consisting of PG, PTMC, and dithiolaned PTMC to formulate multifunctional polymersomes via a “one-pot” strategy. The potent anticancer drug DM1 was covalently anchored inside vesicles during the self-assembly and self-cross-linking of the block copolymer. To target HER-2-positive cancer, postengineering of the ligand trastuzumab to the surface of polymersomes was carried out by click chemistry (Scheme 1). In the present study, the polymersome Tra-PS-DM1 shows high DM1 encapsulation efficacy (>98%), fast release in the redox environment, and minimal leakage (<5%). Moreover, Tra-PS-DM1 presents greatly enhanced cellular uptake in 2D-cultured HER-2-positive cancer cells and remarkable growth inhibition toward HER-2-positive tumoroids. However, we did not observe any efficacy of Tra-PS-DM1 in HER-2-negative tumoroids.

## 2 Experimental Section

### 2.1 Materials and Characterization

Details of materials and instruments applied in this study are described in the Supporting Information.

### 2.2 Synthesis of lPG-PTMC-PDTC

As illustrated in the scheme, an amphiphilic triblock copolymer was obtained by ring opening polymerization of TMC initiated by azide-PEEGE-OH (*M*_n_ = 10 kDa). Azide-PEEGE, TMC, and DTC were synthesized similarly according to the previously introduced protocol.^36^ Typically, stock solutions of organo-catalyst TBD (0.1 mM, 1 mL) in dry DCM and azide-PEEGE-OH (0.1 mM, 2 mL) in dry DCM were prepared under inert flow. With respective ratio, a solution of TBD (0.2 mL) and azide-PEEGE-OH (1 mL) was added to the reactor with TMC (728.91 mg, 7.14 mmol, 1.05 equiv) dissolved in anhydrous DCM (10 mL) under stirring. The reaction proceeded at 50 °C for 24 h. Afterward, predissolved DTC (201.91 mg, 1.05 mmol, 1.05 equiv) in dry DCM was injected to the mixture under vigorous stirring. The polymerization was quenched after 30 min with methanol (0.1 mL). Then, the crude product was precipitated in cold hexane (100 mL), centrifuged, and repeated 3 times. Afterward, the precipitates were filtered and washed with cold hexane (3 × 10 mL). White solids of azide-PEEGE-PTMC-PDTC were finally obtained by vacuum drying.

Subsequently, deprotection of the PEEGE block was carried out by addition of oxalic acid to azide-PEEGE-PTMC-PDTC dissolved in an equally mixed acetone (3 mL) and DI water (3 mL) solution. The hydrolysis reaction proceeded for 4 h at r.t. Afterward, the mixture was dialyzed (MWCO = 2 kDa) against methanol for 24 h. The copolymer azide-lPG-PTMC-PDTC was collected after vacuum drying. Yield: 78%. ^1^H NMR revealed the molecular weight of 14 kDa (Figure S1). The GPC measurement shows that the PDI of obtained lPG-PTMC-PDTC is 1.48 and *M*_n_ is 13.2 kDa (Figure S2).

### 2.3 Fabrication of lPG-Shelled Polymersome Loaded with DM1 and Functionalized by Trastuzumab (Tra-PS-DM1)

Common nanoprecipitation is utilized to formulate PG polymersome loaded with DM1. To 900 μL of phosphate buffer (PB, pH 7.5, 10 mM) was slowly added a 100 μL dimethylformamide (DMF) solution of lPG-PTMC-PDTC (10 mg/mL) and DM1 (1.3 mg/mL) under stirring at 500 rpm. After setting still for 1 h, the solution containing polymersome-DM1(PS-DM1) was dialyzed extensively (MWCO = 3.5 kDa) against PB buffer for 24 h, followed by incubation at 37 °C for 4 h. Afterward, the yielded PS-DM1 (1 mg/mL) was reacted with Tra-BCN (trastuzumab-(1*R*,8*S*,9s)-bicyclo[6.1.0]non-4-yn-9-ylmethyl succinimidyl carbonate) (0.1 mg/mL) at 37 °C overnight. Later, fast protein liquid chromatography (FPLC) was applied for purification of Tra-PS-DM1 and determination of Tra content on the surface of polymersome.

The size and polydispersity of PS-DM1 and Tra-PS-DM1 were measured by dynamic light scattering (DLS). Mimicking the blood circulation, the stability of Tra-PS-DM1 against 100-fold dilution was determined by DLS. Furthermore, the redox-responsiveness of Tra-PS-DM1 was investigated via size change under glutathione (GSH, 10 mM) treatment.

### 2.4 Determination of DM1 Encapsulation Efficacy and DM1 Release Behavior of Tra-PS-DM1

Amounts of payloads collected from the dialysis supernatant were determined by high-performance liquid chromatography (HPLC) or UV/vis spectroscopy. DLC (drug loading content) and DLE (drug loading efficiency) were calculated according to formulas shown as follows:
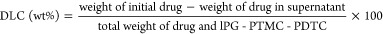

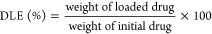


The release behavior of Tra-PS-DM1 was compared with that of the polymersome physically loaded with doxorubicin (Tra-PS-DOX). As an instance, 0.5 mL of freshly constructed Tra-PS-DM1 (1 mg/mL) was placed in mini-dialysis kit (MWCO = 3.5 kDa) and incubated individually with 10 mM GSH (pH 7.4, 25 mL) and 10 mM PB buffer (pH 7.4, 25 mL) at 37 °C. Every 2 h, 5 mL of supernatant was taken and replaced with fresh respective medium. The collected supernatant was then lyophilized. The DOX content released into the supernatant was determined by a UV/vis spectrometer, and DM1 amounts were characterized by HPLC. All the results were obtained from triplicate experiments.

### 2.5 Determination of the HER-2 Expression Level of Breast Cancer Cells

Human breast cancer cells SKBR-3, BT474, and MCF-7 were seeded into a six-well plate at a density of 1 × 10^6^ cells per well and cultivated for 24 h. After detachment, wash, and centrifugation, the cells were resuspended into 100 μL of phosphate-buffered saline (PBS). Then, 10 μL of anti-HER-2 antibody-FITC was added to each cell suspension followed by incubation at 4 °C for 30 min. Later, the cells were washed with PBS (3 × 5 mL) and dispersed into 1 mL of PBS for flow cytometry analysis. For each cell line, at least 1 × 10^4^ cells were analyzed and the results were processed by FlowJo software.

### 2.6 Intracellular Uptake of Tra-PS-DM1 Evaluated by Confocal Laser Scanning Microscopy (CLSM)

Essentially, Tra-PS-DM1 was labeled by Cy5 and confocal laser scanning microscopy (CLSM) was used to examine the intracellular uptake of labeled Tra-PS-DM1 by HER-2-positive SKBR-3, BT474, and HER-2-negative MCF-7 cells. Typically, cells were planted to 8-well ibidi plates at the density of 5 × 10^4^ cells per well and set at 37 °C for 24 h. Following separate addition of Tra-PS-DM1 (1 mg/mL, 10 μL) and PS-DM1 (1 mg/mL, 10 μL) to three cell lines, an additional 4 h of incubation at 37 °C was performed. The medium in each well was aspirated, and cells were gently washed with PBS three times. Then, the treated cells were fixed with 4% paraformaldehyde for 30 min and followed by three times wash with PBS. Afterward, the cells were treated with 0.5% Triton X-100 for 20 min and washed with PBS for another three times. 4,6-Diamino-2-phenylindole (DAPI) was used for cell nuclei staining, and phalloidin-FITC was employed for cell actin staining. Subsequently, cells were washed with PBS five times, and fluorescence images of stained samples were taken by CLSM.

### 2.7 Assessment of Cell Cytotoxicity via CCK-8 Assay

MCF-7, SKBR-3, and BT474 cells were planted to 96-well plates at a density of 5000 cells per well and incubated at 37 °C overnight. The medium was aspirated and replaced with 80 μL of a fresh medium. To each well, 20 μL of Tra-PS-DM1 was added with modulated DM1 concentration from 0.001, 0.005, 0.01, 0.05, 0.1, 0.5, 1.0, 5.0, and 10 μg/mL. After 4 h of co-incubation, the medium from each well was substituted with fresh medium followed by another 68 h of incubation. Ten microliters of Cell Counting Kit-8 (CCK-8) was added to each well and incubated for 2 h. The absorbance at 460 nm of each well was measured by a microplate reader, and corresponding cell viability (%) was calculated relative to the control treated with PBS only.

### 2.8 Spheroid Formation

MCF-7 and BT-474 cells were rinsed with PBS, dissociated into single cells with TrypLE, centrifuged, and resuspended to a density of 2 × 10^4^ cells/mL in RMPI 1640 and DMEM, respectively. Cell density was quantified with Luna cell counters. Ultralow-attachment, U-shaped-bottom 96-well microplates were used for spheroid formation; 100 μL of the cell suspension mentioned above was added to each well. Approximately 2000 cells were allocated in each well to form a single spheroid. The culture medium was changed every other day.

### 2.9 Treatment Efficacy for Tumor Spheroids

To each well containing a single tumoroid, the medium was aspirated and washed with PBS twice. One hundred microliters of Tra-PS-DM1, PS-DM1, and DM1 was added to each well with the IC_50_ concentration screened by 2D modeling. After overnight incubation, PBS was added to each well, and each tumoroid was carefully washed twice. The treated tumoroids were transferred to a 96-flat-well plate individually for Live/Dead staining assay and acid phosphate assay. Briefly, tumoroids were stained with 1 μM calcein AM and 3 μM EthD-III and imaged by fluorescence microscopy. The size of tumoroids was measured from images acquired in a bright field. For APH assay, 5 tumoroids treated with the same group were combined in one well. PBS was discarded to final volume of 50 μL, and 50 μL substrate solution was added to each well. Then 300 μL of standard solution, positive control containing control enzyme, and blank control were prepared. The plate was incubated for 90 min at 37 °C with 5% CO_2_. Eventually, a stop solution was added except for the well of standard solution to terminate the reaction, and the absorbance of each well at 405 nm was recorded by a plate reader. Moreover, treated tumoroids were fixed, permeabilized, and stained with Alexa Fluor 568 phalloidin and Hoechst 33342 for integrity observation by CLSM.

## 3 Results and Discussion

### 3.1 Preparation and Characterization of Tra-PS-DM1

A copolymer of azide-lPG-PTMC-PDTC was obtained via metal-free ring opening polymerization of TMC and DTC. PEEGE with a terminal hydroxyl group was employed as a macroinitiator. ^1^H NMR has shown the desired molecular weight of PTMC and PDTC blocks, and a polydispersity of lPG-PTMC-PDTC of 1.48 was obtained by gel permeation chromatography (GPC) (Figure S2). Azidation of the lPG end group was verified by a Fourier transform infrared (FTIR) spectrum (Figure S3).

Tra-PS-DM1 was fabricated from the lPG-PTMC-PDTC (*M*_w_ = 14 kDa) copolymer via nanoprecipitation. The anticancer drug DM1, presenting a thiol group, was covalently encapsulated into the polymersome’s hydrophobic membrane during self-assembly. Meanwhile, dithiolane ring opening polymerization, triggered by thiol-disulfide exchange, caused the self-cross-linking of the polymersome shell. Trastuzumab modified with a BCN linker was decorated onto the constructed DM1-loaded polymersome through the strain-promoted azide alkyne cycloaddition (SPAAC). Free trastuzumab content was purified by fast protein liquid chromatography (FPLC). The trastuzumab peak was integrated, and the attachment conversion of trastuzumab was 23.8% calculated by comparison with the initial amounts of trastuzumab (Figure S4).

**Figure 1:**
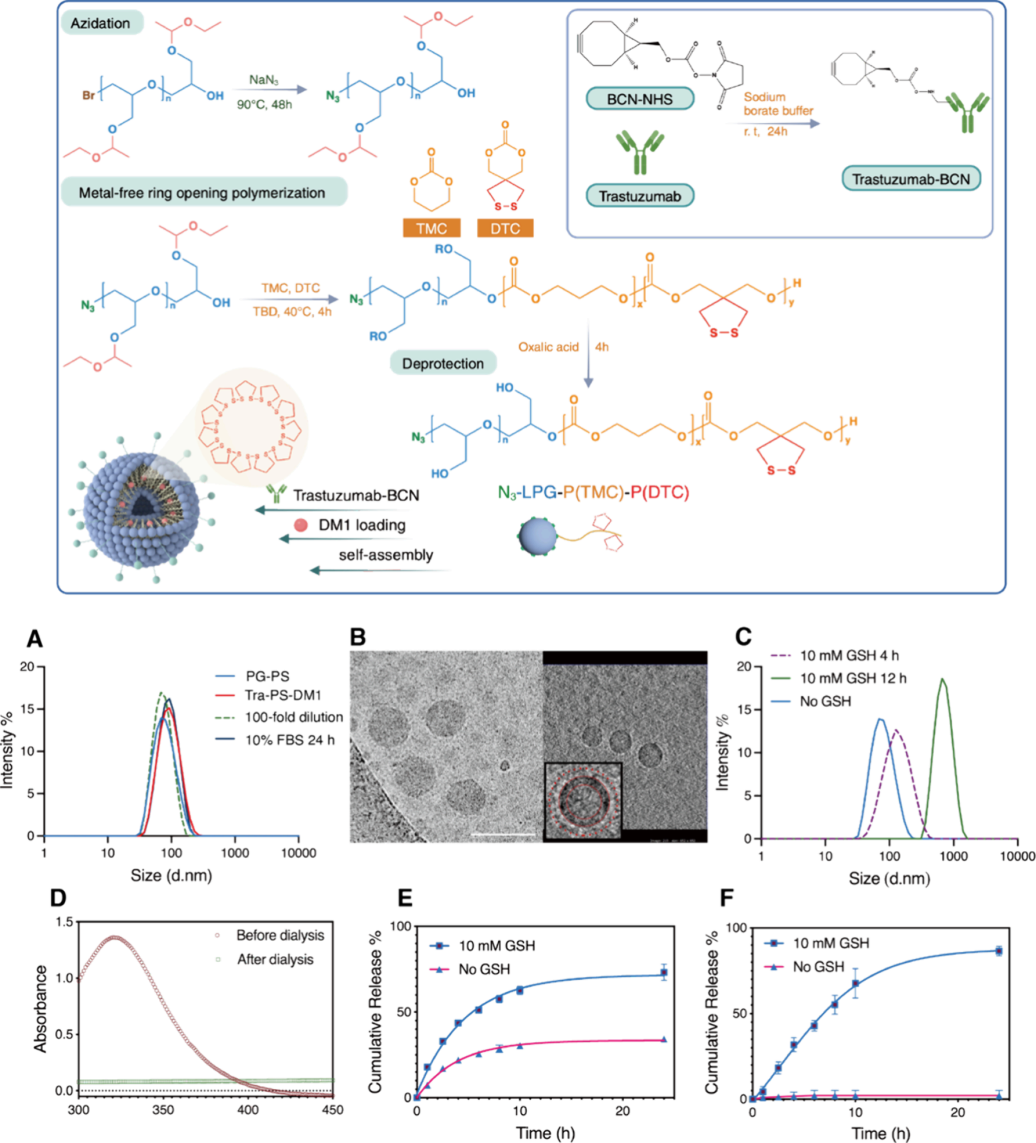
Characterization of PG-PS and Tra-PS-DM1. (A) Size distribution and colloidal stability against dilution measured by DLS. (B) Cryo-TEM images of PG-PS (left) (scale bar, 100 nm) and cryo-electron tomography (right): single slice of the reconstructed 3D volume. (C) Size change of Tra-PS-DM1 after 4 and 12 h of incubation with a reducing agent. (D) Self-cross-linking of the dithiolane ring triggered by DM1 loading. Comparison of the in vitro release of Tra-PSDOX (E) and Tra-PS-DM1 (F).

The dynamic size of PS-DM1, as well as that of Tra-PS-DM1, was characterized by dynamic light scattering (DLS) (Figure 1A). After decoration with trastuzumab, the mean size of polymersome increased slightly from 91.9 to 95.3 nm. Polymersomes remained intact against 100-fold dilution as well as incubation with 10% FBS for 24 h, with a minor size change detected by DLS measurement. Notably, in the presence of 10 mM GSH, the polymersome tends to swell to over 600 nm in diameter after 12 h (Figure 1C). The swelling behavior results from the decrosslinking of internal disulfide networks by GSH. The uniform spherical structure of the constructed polymersomes was observed with cryogenic transmission electron microscopy (cryo-TEM). Additionally, the vesicle framework was investigated by cryo-TEM tomography. The aqueous lumen exhibited similar contrast to the water environment, and the polymeric membrane displayed an average thickness of 8 nm (Figure 1B).

From the UV spectra (Figure 1D), the absorbance of the dithiolane ring at 330 nm disappeared after workup, indicating the thiol-disulfide-triggered self-cross-linking behavior and covalent loading of DM1. HPLC analysis of Tra-PS-DM1 revealed a high DLC of 12.8 wt % (theoretically 13 wt %) and corresponding DLE% of 98.67% (Table 1). Comparing the reduction-stimulated release of doxorubicin (DOX) and DM1, Tra-PS-DM1 exhibited negligible leakage below 5% without a reducing agent (Figure 1F). By comparison (Figure 1E), noncovalently loaded DOX showed moderately higher leakage (above 25% in PB buffer). Upon high reduction sensitivity, rapid and constant release was observed in Tra-PS-DM1 treated with 10 mM GSH, mimicking the cytosol of tumor cells. In contrast to Tra-PS-DOX, the distinguished absolute release content of 84% was achieved with Tra-PS-DM1.

**Table 1:** Size Characterization Using DLS and Loading Efficiency of Tra-PS-DM1

			DLC (wt %)		
	size (nm)	PDI	theory	determined	DLE %
Tra-PS-DMI	95.3	0.17	13	12.9	99.3

### 3.2 HER-2 Targetability and Antitumor Performance of Tra-PS-DM1 in 2D Cell Culture

**Figure 2:**
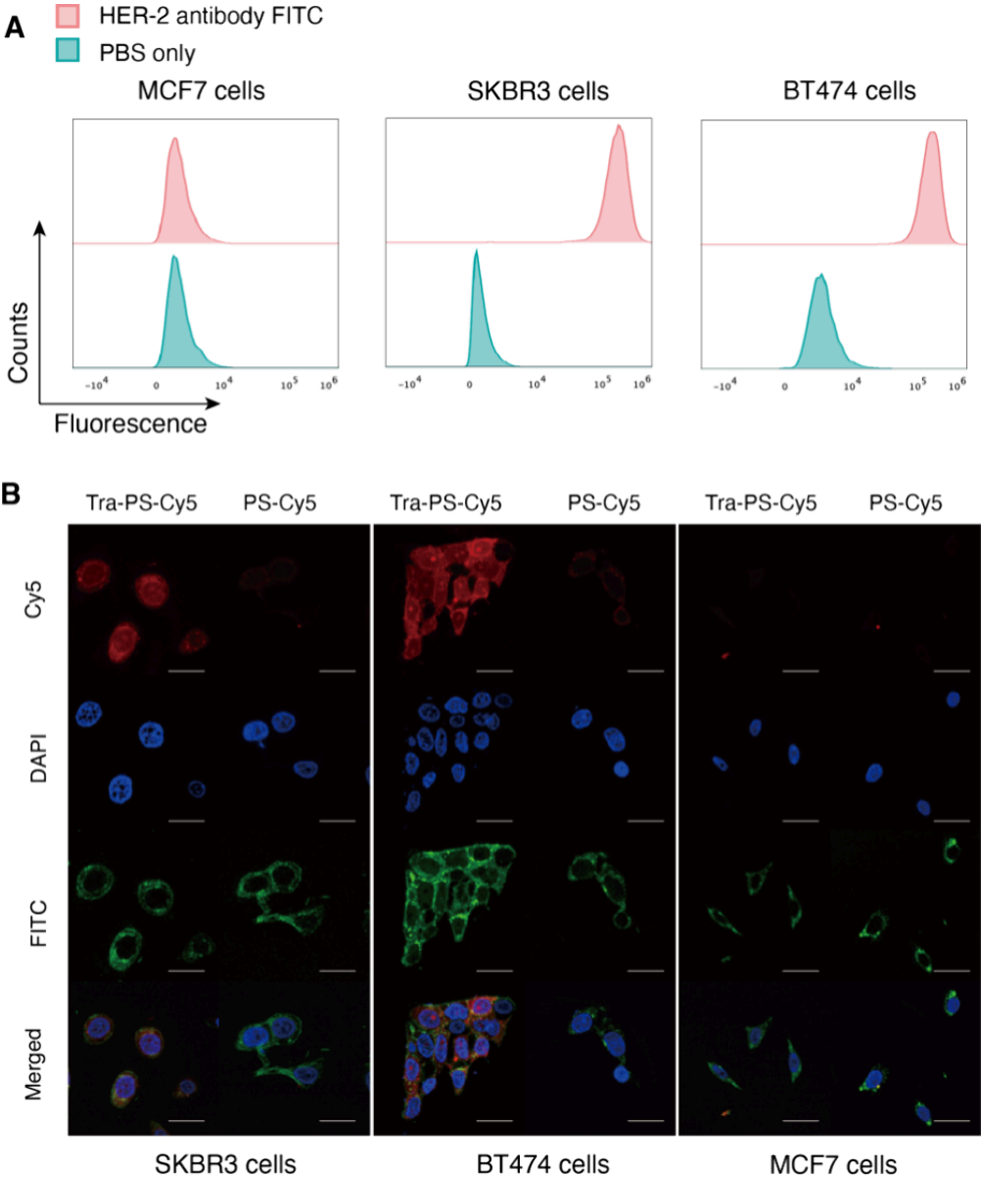
(A) Flow cytometry analysis of the HER-2 expression level of MCF-7, SKBR-3, and BT474 cells (data were processed by FlowJo software). (B) CLSM images of SKBR-3, BT474, and MCF-7 cells incubated with Tra-PS-Cy5 and PS-Cy5 for 4 h (scale bars, 50 μm). Cy5 (red): polymersomes; DAPI (blue): cell nuclei; FITC (green): cell cytoskeleton.

Previous studies have identified BT474 and SKBR-3 cells as HER-2-positive cell lines and MCF-7 cells as HER-2-negative cell lines.^37,38^ Our results for cells labeled with the anti-HER-2-antibody-FITC conjugates shown below (Figure 2A) confirm the high expression of HER-2 in BT474 and SKBR-3 cells and poor HER-2 expression in MCF-7 cells. Therefore, MCF-7 cells were treated as a negative control in subsequent experimentation. CCK8 assays were performed on A549, HeLa, BT474, and MCF-7 cancer cells to assess the cell cytotoxicity of the PG polymersome and Tra-PS. Both PG polymersome and Tra-PS showed high cell compatibility, even at a high concentration of 5 mg/mL (Figure S5).

Cellular uptake and intracellular drug delivery performance among HER-2-positive and HER-2-negative cell lines were evaluated by confocal laser scanning microscopy (CLSM) after individual incubations with Tra-PS-Cy5 and PG-Cy5 for 4 h (Cy5 was encapsulated as a fluorescent indicator). According to the obtained images (Figure 2B), Tra-PS-Cy5 accumulated inside SKBR-3 and BT474 cells, which are HER-2-positive. However, both SKBR-3 and BT474 cells, when treated with PS-Cy5, displayed poor Cy5 fluorescence. Weak Cy5 fluorescence was also observed in MCF-7 cells incubated with Tra-PS-Cy5 and PS-Cy5. Due to the targetability of trastuzumab and the rapid release of payloads triggered by the reducing environment of tumor cells, the uptake of Cy5 was significantly enhanced among HER-2-positive cells. The low fluorescence intensity displayed in HER-2-negative cells, and in the nontargeting PS-Cy5-treated HER-2-positive cells, illustrates the poor uptake efficiency without effective targeting. Furthermore, nonleakage of free dye evidenced by low fluorescence shown in the nontargeted MCF-7, BT474, and SKBR-3 tumor cells treated with nontargeting PS-Cy5 demonstrates that the cargo release was precisely controlled and took place only under reductive conditions.

**Figure 3:**
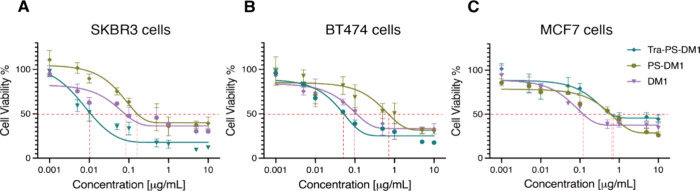
Cell viability of SKBR-3 cells (A), BT474 cells (B), and MCF-7 cells (C) following 4 h of treatment of Tra-PS-DM1, PSDM1, and DM1 (IC_50_ is indicated).

We then evaluated the cell viability after the incubation with Tra-PS-DM1, PS-DM1, and DM1 to assess their antitumor abilities and quantified their IC_50_ correlatively (Figure 3). Notably, in treating SKBR-3 cancer cells, Tra-PS-DM1 exhibited an IC_50_ of 0.011 μg/mL, which is nearly 17-fold lower than that of bold DM1 and 70-fold lower than nontargeted PS-DM1. For BT474 cancer cells, Tra-PS-DM1 showed IC_50_ about 2-fold lower than free DM1 and approximately 15-fold lower than the nontargeted PS-DM1 treatment (Table 2). The rather similar IC_50_ values of native DM1 reveal its consistent levels of antitumor activity across HER-2-positive and HER-2-negative cells. It is evident that the trastuzumab-functionalized polymersome-DM1 complex selectively targets HER-2-positive cancer cells with a high efficacy and potent cytotoxicity.

**Table 2:** IC_50_ Values of DM1, PS-DM1, and Tra-PS-DM1 in BT474 and MCF-7 Cell Lines (Based on DM1 Concentration, μg/mL)

	free DM1	PS-DM1	Tra-PS-DM1
SKBR-3	0.174	0.687	0.0108
BT474	0.119	0.756	0.051
MCF-7	0.133	0.639	0.642

### 3.3 HER-2 Targetability and Antitumor Efficacy of Tra-PS-DM1 in a 3D Multicellular Tumor Spheroid Model

As is well known, HER-2 homodimers, mainly formed in multicellular spheroids, can greatly activate HER-2-mediated signal transduction.^39^ More importantly, HER-2 is demonstrated to be overexpressed predominantly in a 3D-cultured BT474 cell line, similar to breast tumor cells in vivo.^40,41^ Therefore, we further investigated antitumor activities of Tra-PS-DM1 in 3D tumoroids, which have been authorized as a substitute to the animal model due to high similarity to tumors in humans.

**Figure 4:**
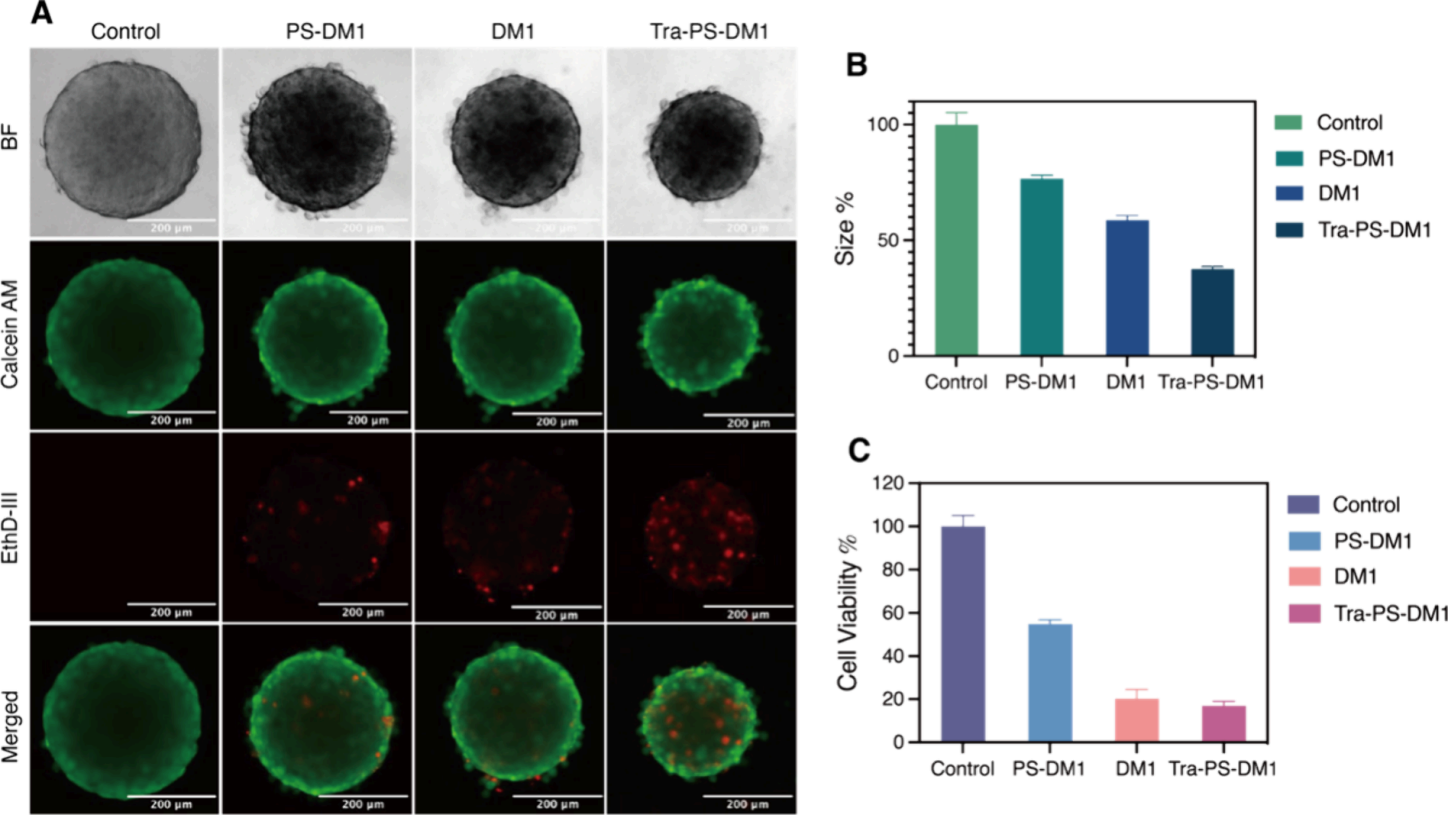
(A) Fluorescence microscopy images of BT474 tumoroids treated with PS-DM1, DM1, and TraPS-DM1 overnight (scale bars, 200 μm). (B) Size analysis of BT474 tumoroids after treatment (the diameter of 5 tumoroids from the same group was measured with ImageJ, and size % is calculated by size % = *d*^3^/*d*_control_^3^). (C) Cell viability of BT474 tumoroids determined by APH assay.

**Figure 5:**
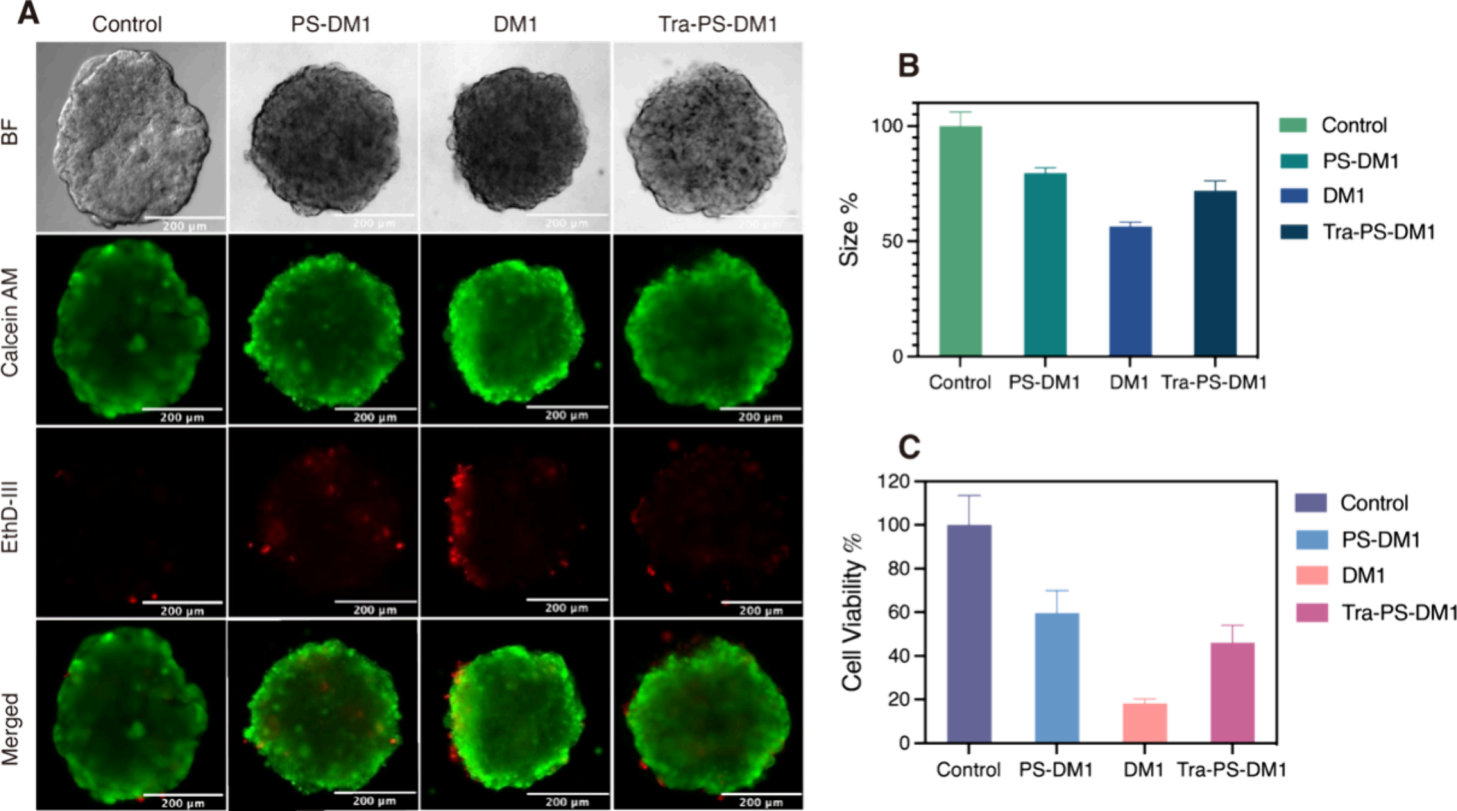
(A) Fluorescence microscopy images of MCF-7 tumoroids treated with PS-DM1, DM1, and Tra-PSDM1 overnight (scale bars, 200 μm). (B) Size analysis of MCF-7 tumoroids after treatment (the diameter of 5 tumoroids was measured in each group by ImageJ, and size % is calculated by size % = *d*^3^/*d*_control_^3^). (C) Cell viability of MCF-7 tumoroids determined by the APH assay.

**Figure 6:**
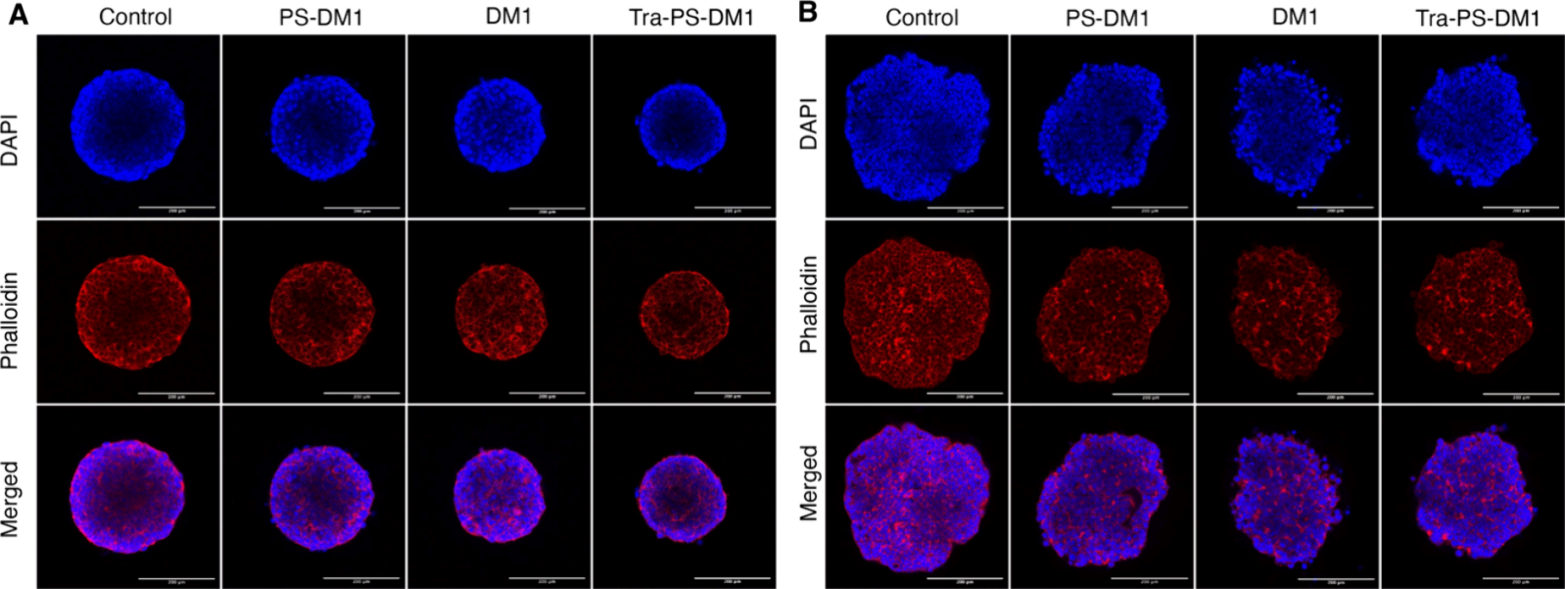
CLSM images of BT474 cells (A) and MCF-7 cells (B) in a 3D culture. Tumoroids were treated with PS-DM1, DM1, and Tra-PS-DM1 at DM1 concentration of IC_50_ obtained from 2D culture for 12 h, then fixed, and stained for microscopy (scale bars, 200 μm).

As indicated in Figure 4, PS-DM1, DM1, and Tra-PS-DM1 have shown potency toward both BT474 tumoroids and MCF-7 tumoroids. However, the efficacy differs between groups. Bright field imagery (Figure 4A) shows the remarkable size shrinkage of Tra-PS-DM1-treated BT474 tumoroids. In contrast, DM1-treated MCF-7 tumoroids were found to exhibit the greatest size reduction among MCF-7 tumoroids. In addition, we performed a statistical study of tumoroid size on 5 tumoroids per group. As illustrated clearly in Figure 4B, the size of BT474 tumoroids treated with Tra-PS-DM1 decreased by up to 62%. This result is in contrast to respective shrinkages of 25 and 44% from nontargeted PS-DM1 and bold DM1 drug. The size of the MCF-7 tumoroids treated with DM1 (Figure 5B) was reduced by about 46%, which aligns with BT474 tumoroids treated with DM1. Due to the weak overexpression of HER-2, PS-DM1 and Tra-PS-DM1 present rather similar inhibitory potential to the progression of MCF-7 tumoroids. In addition to size shrinkage, the fluorescence of stained dead cells in BT474 tumoroids was found to intensify from PS-DM1 to DM1 and from DM1 to Tra-PS-DM1 (Figure 5A). In the case of MCF-7 tumoroids, the DM1-treated group showed the strongest such increase caused by efficient passive diffusion. Notably, we notice that the dead cells mostly were distributed on the surface of tumoroids after treatment, especially on BT474 tumoroids due to HER-2-associated cell–extracellular matrix interaction. However, we observed the dead cells only in the center of BT474 tumoroids after treatment with Tra-PS-DM1, which strongly demonstrates the potent penetration of Tra-PS-DM1 in the 3D multicellular model. Furthermore, the cell viability of tumoroids was quantified by the APH assay. The obtained results are displayed in Figures 4C and 5C. Consistent with size reduction and the number of dead cells, BT474 tumoroids treated with Tra-PS-DM1 presented the lowest cell viability around 16%, followed by the DM1-treated group (20%) and PS-DM1-treated tumoroids (50%). As expected, the lowest cell viability for MCF-7 spheroids was observed in those spheroids incubated with the DM1 drug: approximately 18% compared to the control treated with PBS buffer. It is therefore evident that Tra-PS-DM1 can selectively target HER-2-positive cells with extraordinary affinity and cytotoxicity. Treated tumoroids were also stained with Alexa Fluor 568 phalloidin and Hoechst 33342 to characterize cell integrity and packing behavior. As expected from results up to this point, in MCF-7 tumoroids, we observed distinct damage to the extracellular matrix along with the surface of tumoroids (Figure 6B). The cell density, especially on the surface of MCF-7 tumoroids after DM1 treatment, diminished significantly. Intriguingly, BT474 tumoroids displayed minor differences in integrity and surface regulation after treatment, confirming the crucial role of HER-2 in cell adhesion and proliferation (Figure 6A). The rapid regeneration of the proliferation zone of BT474 tumoroids has mainly disrupted the accessibility of drug content in the tumor microenvironment. However, the significant shrinkage of HER-2-positive tumoroids after treatment with Tra-PS-DM1 in all dimensions demonstrates that the trastuzumab-functionalized polymersome efficiently enhances cellular DM1 uptake and induces constant DM1 release upon targeted sites.

## 4 Conclusions

In conclusion, robust polymersome-DM1 conjugates were developed exclusively with a polyglycerol shell as a new PEG alternative. This multifunctional polymersome, formulated via a facile “one-pot” approach, showed exceptional stability against dilution and 10% FBS incubation. Through self-crosslinking induced by DM1, loading efficiency was significantly enhanced, and payload leakage was minimized. The polymersome decorated by trastuzumab accomplished systematic targetability toward HER-2-positive cancer in both 2D and 3D cultures. With a single treatment in 24 h, the size of HER-2-positive tumoroids was reduced up to 62%. Notably, the lack of toxicity of polymersome may make it suitable as a highly biocompatible platform for diverse cargos. The polymersome described here suggests a promising treatment avenue for HER-2-positive cancer.

## Supporting Information Available

Details of materials, characterization, ^1^H NMR spectrum, GPC chromatograph of lPG-PTMC-PDTC, FPLC chromatograph of Tra-PS-DM1, and cell cytotoxicity test on A549, HeLa, BT474, and MCF-7 cells (PDF)
