# Supplementary material for: Polyglycerol-Shelled Reduction-Sensitive Polymersome for DM1 Delivery to HER-2-Positive Breast Cancer
Source: Biomacromolecules. 2024 Jun 22;25(7):4440–8. doi: 10.1021/acs.biomac.4c00512 (PMC11238329; doi:10.1021/acs.biomac.4c00512)
Supplement: Supplementary file 1 — bm4c00512_si_001.pdf [file bm4c00512_si_001.pdf]

Supplementary information

Polyglycerol-shelled reduction-sensitive polymersome for DM1 delivery to HER-2 positive breast cancer

Guoxin Ma[a], Daniel Braatz[a], Peng Tang[a], Yian Yang[a], Elisa Quaas[a], Kai Ludwig[a], Nan Ma[a,b], Huanli Sun[c], Zhiyuan Zhong[c,]*, Rainer Haag[a,]*

[a]Institut für Chemie und Biochemie, Freie Universität Berlin, Takustr. 3 14195, Germany.
[b]Institute of Active Polymers, Helmholtz-Zentrum HEREON, 14513 Teltow, Germany.
[c]Biomedical Polymers Laboratory, College of Chemistry, Chemical Engineering and Materials Science, and State Key Laboratory of Radiation Medicine and Protection, Soochow University, Suzhou, 215123, PR China.

*Email: zyzhong@suda.edu.cn
*Email: haag@zedat.fu-berlin.de

Materials

Sodium azide, 2, 2-bis(bromomethyl) 1, 3-propanediol, triazabicyclo[4.4.0]dec-5-ene (TBD), Anhydrous dichloromethane (≥ 99.8%, contains 40-150 ppm amylene as stabilizer), anhydrous tetrahydrofuran (≥ 99.9%, inhibitor-free), Anhydrous dimethylformamide (99.8%), Doxorubicin, Pur-A-Lyzer mini dialysis kit (MWCO 6-8 kDa), acid phosphatase assay kit were purchased from Sigma Aldrich (St. Louis, MO, USA). Oxalic acid, dialysis tubes (MWCO 3.5 kDa), hexane and diethyl ether were ordered from VWR chemicals (Radnor, Pennsylvania, USA). Poly-(Ethoxyethyl Glycidyl Ether) (PEEGE) of 20 kDa was synthesized by Daniel Kutifa (Freie Universität Berlin) reported before[1]. Dithiolane functionalized trimethylene carbonate (DTC) and trimethylene carbonate (TMC) were provided by the Group of Prof. Zhiyuan Zhong, Soochow University[2]. Cy5 was purchased from FEW chemicals, Germany. Mertasine (DM1) and Trastuzumab were ordered from MedchemExpress, Sweden. Dulbecco's modified eagle medium, RPMI medium 1640, 0.05% trypsin-EDTA (1X), DPBS buffer and feta bovine serum were purchased from Gibco, USA. Cell counting kit-8, LIVE/DEAD viability /cytotoxicity kit, Alexa-fluor 568 phalloidin, Fluorescein, 4',6-Diamidin-2-phenylindol, Dihydrochlorid (DAPI), anti-human HER2 monoclonal antibody-FITC, 96 U-bottom plates were acquired from Thermo Fischer.

Characterization

The polymer structures were determined by ^{1}H NMR conducted on spectrometer ECX 400 (JEOL, USA) at 400 MHz. The chemical shifts were calibrated against residue solvent signals. The molecular weight and polydispersity index were measured by gel permeation chromatograph (GPC) LC-2030 produced by Shimadzu, Japan. The characterization was performed by DMF with 3 g/L LiBr and 6 g/L AcOH as an eluent at the flow rate of 1 mL/min. The calibration for columns was carried out by series of Polystyrene and sample concentration of 5 mg/mL was required. The diameter, size distribution of polymersomes were determined at 25°C by dynamic light scattering (DLS, Zetasizer ultra, Malvern Panalytical, UK) equipped with a 632.8 nm He-Ne laser beam alongside non-invasive back scattering technology. Specimen preparation for cryogenic transmission electron microscopy (cryo-TEM) was performed by plunge-freezing using an FEI Vitrobot Mark IV (Thermo Fisher Scientific Inc., Waltham, Massachusetts, USA) and cryo-TEM and cryo-electron tomography (cryo-ET) were performed using an FEI TALOS ARCTICA electron microscope (Thermo Fisher Scientific Inc., Waltham, Massachusetts, USA) at an accelerating voltage of 200 kV. For cryo-ET, single-axis tilt series (±64° in 2° tilt angle increment) were acquired with an FEI Falcon 3EC 4k×4k direct electron detector using a Volta phase plate at 28 K primary magnification with a total dose of less than 120 e-/Å^2. The tomograms were reconstructed with binned data (binning factor 2) using ThermoFisher Inspect3D software, version 3.1.0. Purification of Tra-PS-DM1 was performed by fast protein liquid chromatograph (FPLC), ÄKTA pure 25, Cytiva, USA. Concentration of Mertasine in encapsulation and release study was analyzed by high performance liquid chromatography (HPLC), Knauer Smartline, Germany. Concentration of DOX was measured by UV/vis, Agilent Cary, USA. Cells labelled by anti-HER-2-antibody-FITC conjugates were detected by flow cytometry, Attune NxT Thermo Fisher. Absorbance of cells treated with CCK-8 assay and APH assay were measured by microplate reader, Tecan Spark, Switzerland. Imaging of intracellular uptake and tumoroids morphology was conducted by confocal laser scanning microscopy (CLSM), Leica TCS SP8,

Germany. Fluorescence microscope, Zeiss Axis observer Z1 colorcam, Germany was used for Live/dead cell imaging.

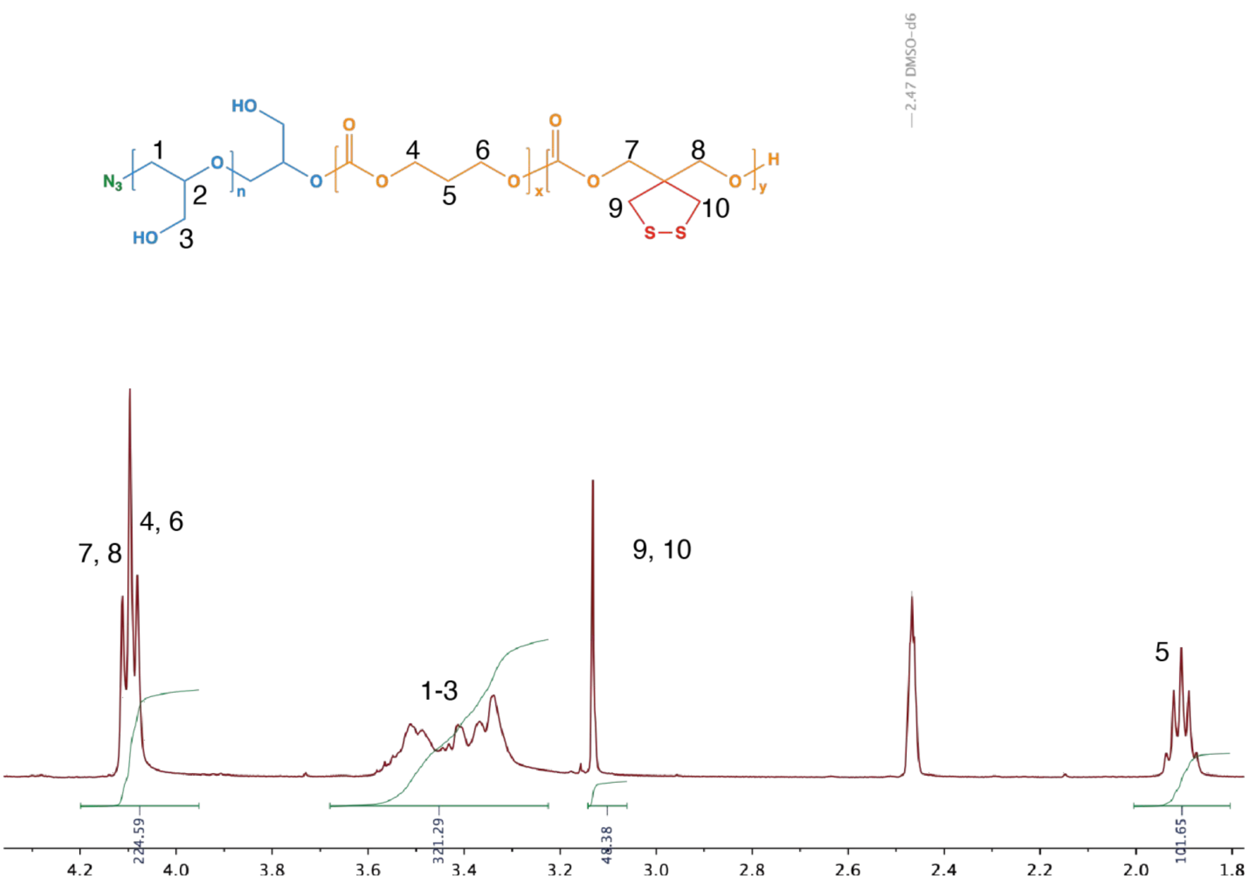

Fig. S1. ^{1}H NMR (400 MHz, DMSO-d_6) of lPG-PTMC-PDTC block copolymer: δ 1.86-1.94 (quint, 2H) and 4.05-4.13 (t, 4H) (PTMC); δ 4.08 (t, 4H) and 3.13 (t, 4H) (PDTC); δ 3.21-3.59 (br, 5H) (lPG backbone). Comparing among the integrals of the signals at δ 1.86-1.94 and 4.05-4.13 (PTMC) and δ 4.08 and 3.13 (PDTC) with δ 3.21-3.59 (lPG), the molar mass of lPG-PTMC-PDTC can be concluded.

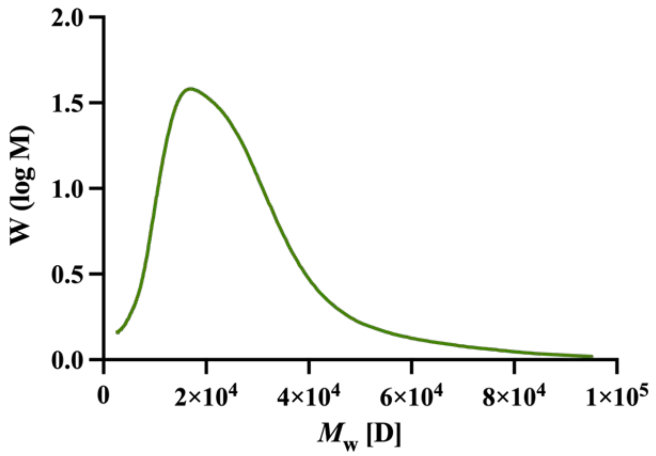

Fig. S2. GPC chromatograph of lPG-P(TMC)-P(DTC) triblock copolymer.

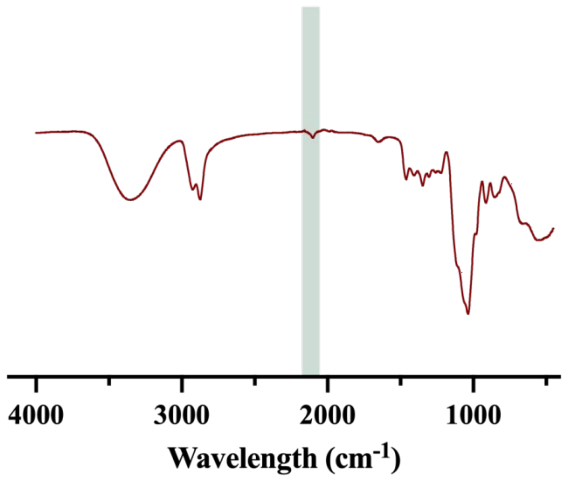

Fig. S3. FTIR spectra of azide functionalized lPG (-N_3 stretch at 2130 cm^{-1}).

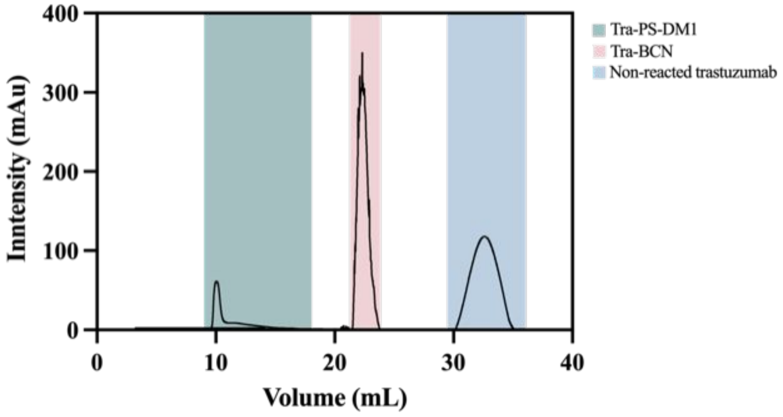

Fig. S4. FPLC chromatograph of Tra-PS-DM1, Tra-BCN, free trastuzumab.

Table S1. Conversion of modification of trastuzumab

	Trastuzumab-BCN	Tra-PS-DM1
Conversion %	51.3	23.8

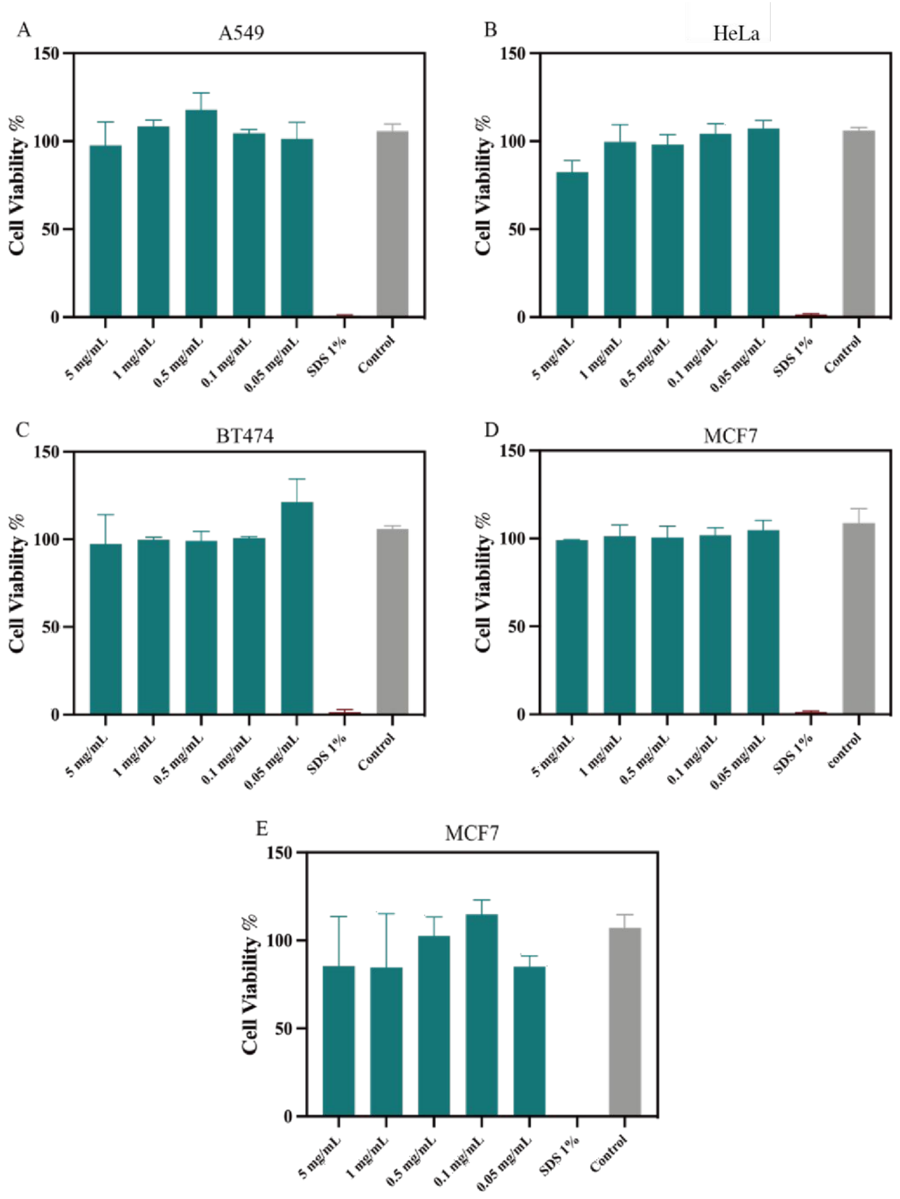

Fig. S5. Cell cytotoxicity of blank polymerosome (PS) tested on various cell lines (A) A549 cells, (B) HELA cells, (C) BT474 cells, (D) MCF7 cells. (E) Cell cytotoxicity of Tra-PS characterised on HER2-nagative MCF7 cells.

Reference

(1) Pouyan, P.; Nie, C.; Bhatia, S.; Wedepohl, S.; Achazi, K.; Osterrieder, N.; Haag, R. Inhibition of Herpes Simplex Virus Type 1 Attachment and Infection by Sulfated Polyglycerols with Different Architectures. *Biomacromolecules* **2021**, *22* (4), 1545-1554.

(2) Wei, J.; Meng, H.; Guo, B.; Zhong, Z.; Meng, F. Organocatalytic ring-opening copolymerization of trimethylene carbonate and dithiolane trimethylene carbonate: Impact of organocatalysts on copolymerization kinetics and copolymer microstructures. *Biomacromolecules* **2018**, *19* (6), 2294-2301.
